# Therapeutic Branding: A Common and Bizarre Practice That Scars for Life

**DOI:** 10.7759/cureus.87123

**Published:** 2025-07-01

**Authors:** Shaurya Tewari, Mahadev Meena, Tarini Prasad Dandasena, Yasmee Khan, Rajnish Joshi, Ankur Joshi

**Affiliations:** 1 Internal Medicine, All India Institute of Medical Sciences, Bhopal, Bhopal, IND; 2 General Medicine, All India Institute of Medical Sciences, Bhopal, Bhopal, IND; 3 Community and Family Medicine, All India Institute of Medical Sciences, Bhopal, Bhopal, IND

**Keywords:** bizarre, branding, chronic liver disease, rod, scars

## Abstract

Introduction: Branding of skin in a living person is an ancient practice, was used to indicate ownership, as a membership of a cult, as a method of punishment, and even for public humiliation. While the above uses of branding are on a decline, we have come across numerous instances of the use of branding as a therapeutic measure. These archaic practices exacerbate the original illness and lead to complications such as secondary infection, scars, and keloid formation. Our objectives for this study were to document and analyze the use of therapeutic branding, focusing on its clinical implications, sociodemographic profile of affected individuals, underlying medical conditions for which branding was performed, and association of morbidity and mortality.

Methodology: This is a hospital-based retrospective observational study conducted in the Department of General Medicine at a tertiary care hospital of central India. We identified branding marks in admitted patients in the medical wards, and as part of the standard of care, we documented the history and circumstances leading to branding marks and captured a photograph of these lesions after consent.

Results: We identified branding marks in about 15 admitted patients. Five (33%) of these had an acute medical condition, and two (13%) of them did not survive the hospital stay. Another 10 (67%) had a chronic medical condition, and four (27%) of these did not survive beyond hospital stay. In 10 (67%) patients, branding delayed the diagnosis of the underlying condition.

Conclusions: Therapeutic branding is complicating the underlying illness and increasing mortality and morbidity.

## Introduction

Branding is the act of burning a mark or symbol onto the skin of a living individual. This ancient practice was used to indicate ownership, as a membership of a cult, as a method of punishment, and even for public humiliation. It was also a result of the spectrum of religious beliefs, sociocultural, and pseudoscientific practices. Branding was also used for the treatment of various medical conditions [1]. While the above uses of branding are on a decline, we have come across numerous instances of the use of branding as a therapeutic measure [2-3]. Such a therapeutic use of branding involves a hot iron rod or a coin by faith healers. Third-degree burns, so produced, lead to scars that persist for life. In modern clinical literature, therapeutic branding has been reported only sporadically, primarily in pediatric populations or within the context of cultural studies. However, our study systematically highlights the continued use of therapeutic branding in adult patients for both acute and chronic conditions. It not only underscores the physical harm associated with branding but also documents its clinical consequences, including delayed diagnosis, misdiagnosis, and related complications. Additionally, our study includes photographic evidence and morphological classification of branding lesions aspect which is scarcely addressed in existing literature. It further explores the sociocultural drivers and economic factors that contribute to the persistence of this practice. By referencing Section 324 of the Indian Penal Code, which pertains to voluntarily causing hurt by dangerous means, our study also integrates clinical findings with legal and ethical dimensions, emphasizing the need for a multidisciplinary response. We aim to document the clinical, sociocultural, and legal dimensions of therapeutic skin branding in adult patients with acute and chronic medical conditions, including its patterns, consequences, and underlying drivers.

## Materials and methods

This is a retrospective observational study, and it was conducted in the Department of General Medicine at a tertiary care center of central India over a duration of six months, from June to December 2023. Individuals aged more than 18 years who were admitted with branding marks for either acute or chronic diseases were included. Patients who did not consent to participate in the study were excluded. Ethical clearance was obtained from the institutional ethics committee (IHEC/SR/2024/Apr/27). Informed consent was secured from all patients participating in the study. Medical records were reviewed to identify patients with branding marks. As part of the standard of care, the history and circumstances leading to the branding marks were documented. Consent was also obtained from participants to photograph these lesions during treatment. Representative photographs of the branding lesions were taken and included in the study. The presence of branding marks was the primary variable in this study. Several secondary variables were assessed, including demographic information (age, gender, and occupation), reasons for branding, and associated clinical outcomes. For the statistical analysis, descriptive statistics were employed. The data were summarized primarily through the count and proportion of patients identified with branding marks.

## Results

**Figure 1:**
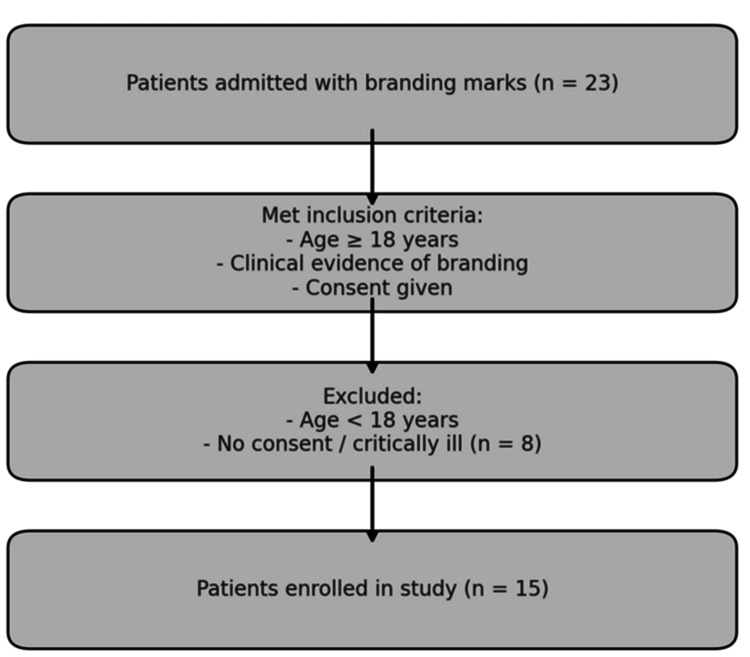
Patient enrollment flowchart. Image credit: Shaurya Tewari.

Figure 1 shows the patient enrollment flowchart.

The details of the 15 cases of branding, along with figure numbering, are presented in Table 1. The age ranged from 21 to 58 years. Of the 15 patients, three (20%) were female and 12 (80%) were male. Occupation and outcome of patients are mentioned in Table 1. Five (33%) of these patients had an acute medical condition, and two (13%) expired during the hospital stay. Another 10 (67%) had a chronic medical condition, and four (27%) of these did not survive beyond the hospital stay. In patients with chronic disease, precise information on the timing of branding was unavailable, as most were unable to recall the exact date.

**Table 1:** Series of cases that presented with branding marks. *These individuals succumbed to their illness during the hospital stay. M, male; F, female

Serial no.	Age/sex; occupation	Type; Figure	Location	Underlying disease	Implications of branding	Outcome
Acute medical conditions (*n* = 5)						
1	25/M; Farmer	Linear; Figure 2A	Forearm	Acute; tetanus	Delayed diagnosis	Cured and discharged
2	26/M; Student	Circular; Figure 2B	Wrist, abdomen	Acute; hepatitis	Delayed diagnosis	Discharged
3	40/M; Policeman*	Semilunar; Figure 2C	Abdomen	Acute; hepatitis	Delayed diagnosis	Death
4	54/F; Homemaker	Circular; Figure 2D	Forearm	Acute stroke	Disability	Discharged
5	58/M; Farmer*	Linear; Figure 2E	Thigh	Acute stroke	Delayed diagnosis	Death
Chronic medical conditions (*n* = 10)						
6	21/M; Student	Irregular; Figure 2F	Hand	Rheumatoid arthritis	Local scar	Discharged
7	22/M; Student	Circular; Figure 3A	Abdomen	Chronic liver disease	Delayed diagnosis	Discharged
8	35/F; Homemaker*	Circular; Figure 3B	Wrist, thorax	Rheumatoid arthritis	Local infection	Death
9	39/M; Laborer	Circular; Figure 3C	Wrist	Rheumatoid arthritis	Local scar	Discharged
10	40/M; Businessman	Circular; Figure 3D	Wrist, abdomen	Chronic liver disease	Delayed diagnosis	Discharged
11	42/M; Businessman*	Irregular; Figure 3E	Abdomen	Chronic leukemia	Delayed diagnosis	Death
12	44/F; Homemaker	Circular; Figure 3F	Abdomen	Chronic liver disease	Delayed diagnosis	Discharged
13	44/M; Guard*	Linear; Figure 4A	Abdomen	Rheumatic heart disease	Delayed diagnosis	Death
14	48/M; Farmer	Circular; Figure 4B	Abdomen	Chronic liver disease	Delayed diagnosis	Discharged
15	49/M; Farmer*	Linear; Figure 4C	Abdomen	Chronic liver disease	Local infection	Death

**Figure 2:**
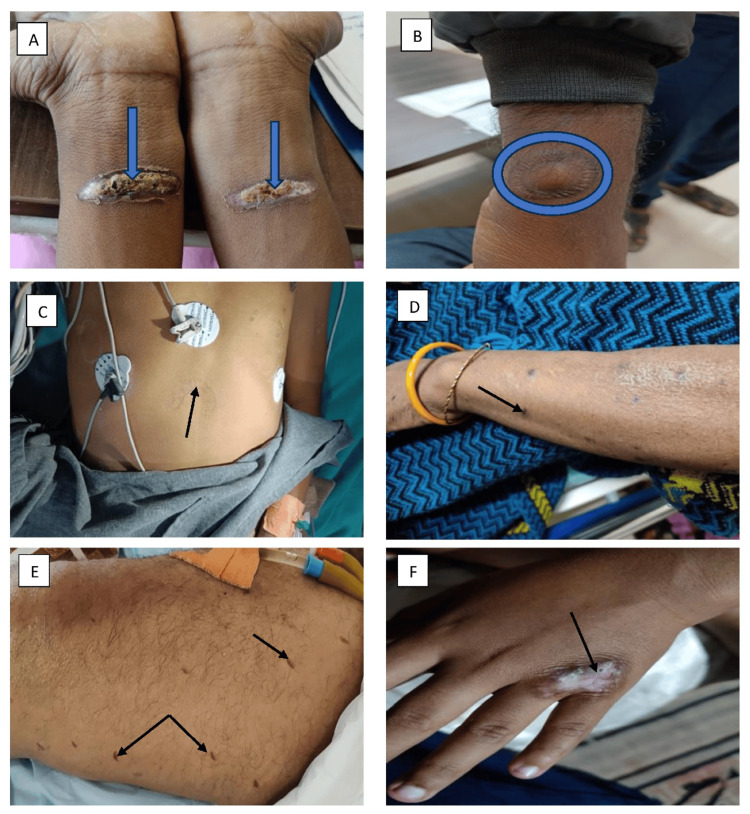
Various branding marks on patients with different medical conditions. (A) Branding over the ventral surface of the forearm in a patient with generalized tetanus (blue arrow), (B) circular branding at the wrist in acute hepatitis A patients (blue ring), (C) branding mark on the abdomen in a patient with acute fulminant liver failure (black arrow), (D) small branding mark on the forearm in a patient with cerebrovascular accident (CVA) (black arrow), (E) multiple branding marks on the thigh in a patient with acute intracranial hemorrhage, aimed at treating neurological weakness (black arrow), and (F) scar hypertrophy at the metacarpophalangeal joint in a patient with adult-onset Still's disease (AOSD) (black arrow).

**Figure 3:**
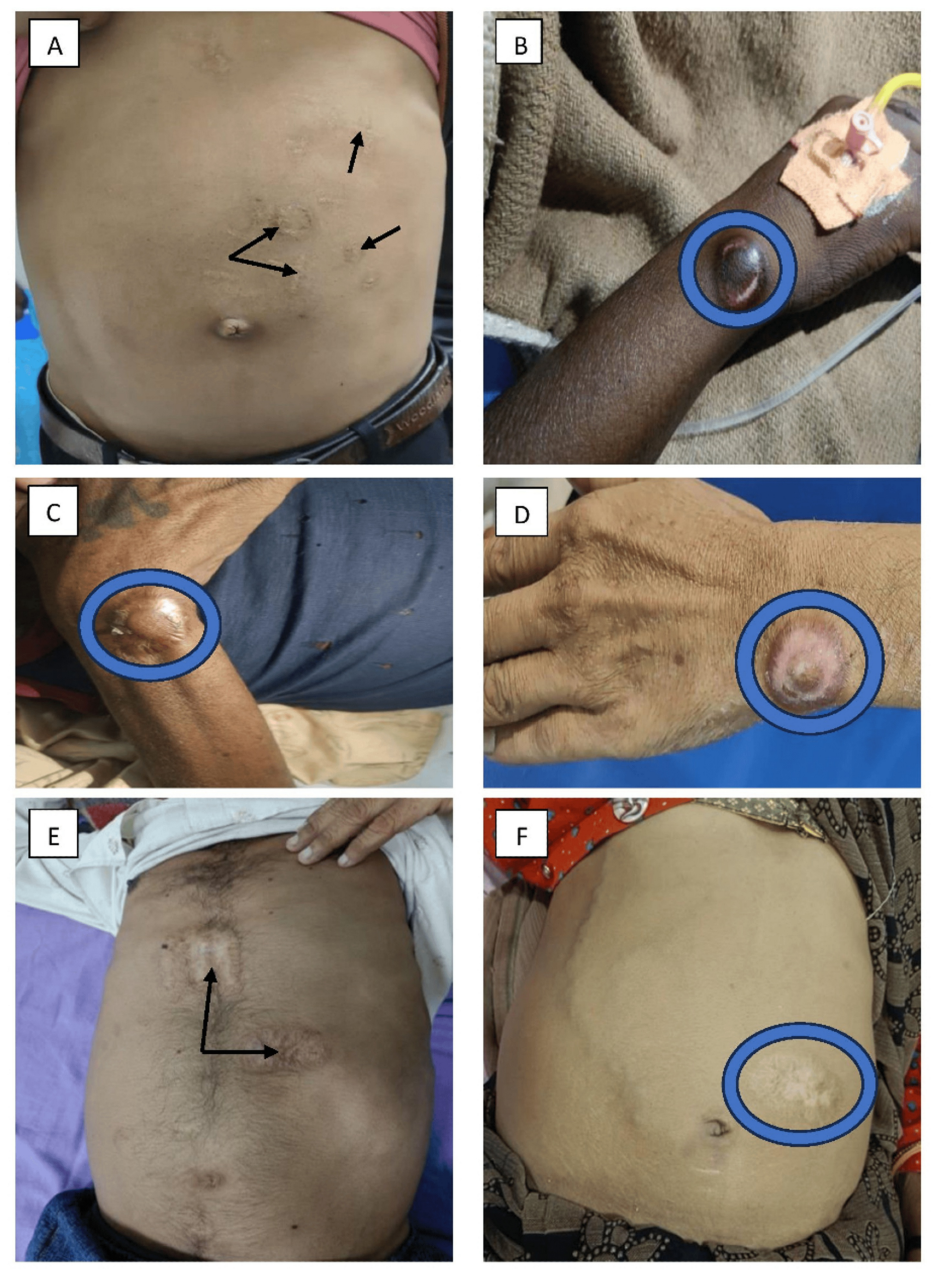
Various branding manifestations on patients with different medical conditions. (A) Multiple-shaped branding marks on the abdomen in a patient with chronic liver disease related to hepatitis B (black arrow), (B) circular branding at the wrist joint in a patient with rheumatoid arthritis (blue ring), (C) circular branding at the wrist joint in a patient with rheumatoid arthritis (blue ring), (D) circular branding at the wrist joint in a patient with chronic liver disease (blue ring), (E) branding scar over the abdomen in a patient with chronic myeloid leukemia (CML), used for the treatment of abdominal discomfort (black arrow), and (F) branding mark over the abdomen in a patient with chronic liver disease (CLD) related to hepatitis B, aimed at treating initial jaundice (blue ring).

**Figure 4:**
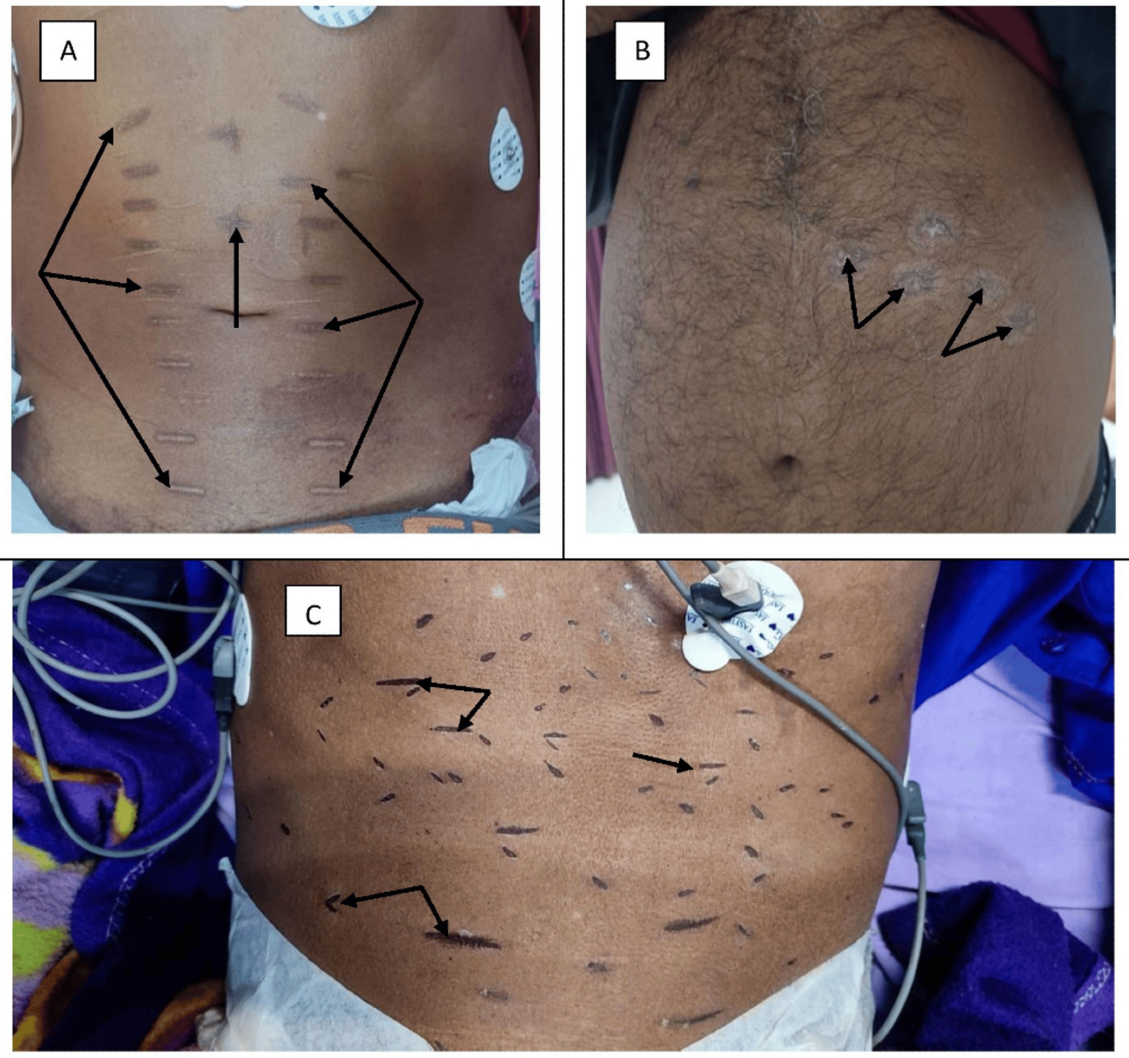
Various branding marks on patients with different medical conditions. (A) Multiple branding scars over the abdomen in a patient with rheumatic heart disease (black arrow), (B) multiple branding marks over the abdomen in a patient with alcohol-related chronic liver disease (CLD), also used to treat initial jaundice (black arrows), and (C) multiple branding lesions on the abdomen in a patient with alcohol-related CLD, utilized to treat abdominal distension.

Eight branding marks were circular (Figures 2B, 2D, 3A-3D, 3F, 4B), four were linear (Figures 2A, 2E, 4A, 4C), two were irregular (Figures 2F, 3E), and one was semilunar (Figure 2C). 

In 10 out of 15 instances, branding led to a delay in the diagnosis of the underlying condition. The practice was advised by ill-informed village individuals and family members of the patients themselves. Branders would assure patients and their relatives that they could cure the disease through this method. Most patients sought subsequent healthcare only when the branding did not have the desired therapeutic effect. In five patients (33.3%), family members resorted to branding as modern medical measures did not lead to a desired improvement in the medical condition. In our study, the majority of patients were male (12, 80%), with only three female patients (20%). Males were observed to have poorer post-branding hygiene practices, often neglecting proper wound care and using unsterile materials for dressing. This may reflect occupational exposure and cultural perceptions of self-care. Consequently, male patients experienced a higher rate of post-branding complications, including secondary infections and delayed wound healing. There were some notable diagnostic uncertainties due to branding. A person with tetanus had burned skin over both wrists. Initially, we considered these as injuries that had induced tetanus. However, detailed history revealed that these were branding injury marks, which the patient got after he had developed symptoms of lockjaw. This led to a change in diagnosis toward cryptogenic tetanus, highlighting the persistence of branding as a treatment method in the community. Another patient with acute stroke, who had hemiplegia, had branding marks over the thigh. A careful history and examination revealed these marks as a result of branding rather than an injury.

The practice results not only in delays but also in a complete denial of care. Three individuals (who had chronic leukemia, rheumatic heart disease, and rheumatoid arthritis) presented to the tertiary care hospital with well-advanced disease. A person with chronic leukemia (Figure 3E) was so assured about the therapeutic effect of branding that he presented to us in blast crisis and expired. The patient who had rheumatic heart disease (Figure 4A) was asked to seek further medical care only when the branding scars had healed. Another patient with rheumatoid arthritis (Figure 3B) had sought therapeutic branding and had not received any medical treatment before presentation; he subsequently succumbed during the current hospital stay. Furthermore, four patients paid for branding services using currency.

## Discussion

Branding techniques are prevalent and often exacerbate the original ailment [2]. In India, branding is employed both in children and adults [3]. There is no clear evidence as to when the practice of branding emerged as a treatment modality. It originated centuries ago, and according to one theory, in ancient times, traditional healers used hot rods to burst septic boils and cauterize infections. However, over time, the practice acquired significance with faith healers. In rural and tribal communities, the practice is associated with local customs, propagating body branding as a cure for common diseases in communities [4].

Branding as a prevention or treatment for many diseases remains a prevalent superstitious practice in rural areas of India. Family members often believe that the offending agent is expelled from the branded site, thereby purging the body of the disease. The most commonly used instrument is the tip of a hot metal rod, although heated nails, wires, incense sticks, and hot bangles may also be utilized. The forehead, face, chest wall, and abdomen are typical sites for branding. Different materials like honey, dung, or even ash are applied to the burned area, potentially leading to infections and fatal sepsis in patients. Some patients with a genetic predisposition for scarification may develop extremely large and cosmetically unappealing keloids, as observed in our patient with adult-onset still disease [2]. These ancient, crude, and inhumane methods carry the risk of complications [5]. Complications from branding include acute infection, transmission of blood-borne pathogens, allergic reactions, and sequelae arising from third-degree burns [6-7]. The practice of applying saliva, ash, or herbal paste to the burn wounds exacerbates morbidity. In Eastern societies where modern medical care is inaccessible to much of the population, patients seek branding treatments for various medical conditions such as backache, sciatica, arthritis, paralysis, facial palsy, ascites, splenomegaly, lymphadenopathy, jaundice, glaucoma, migraine headaches, and sore throat [8].

Branding and scarification result in patterns of permanent scars on the skin, stemming from different procedures such as electroacoustic methods, chemicals, laser treatments, heated metals, or freezing. These procedures increase the risk of local and systemic infections. Various forms of branding exist, including strike branding, hypothermal (freeze) branding, chemical branding, electrocautery branding, and laser branding. Depending on the method used, allergic reactions are possible. Another issue can be hypertrophic scars, which in severe cases could lead to limited mobility of limbs, with effects potentially manifesting years to decades later [9]. In our study, branders advised to avoid medical treatment, citing a high risk of death in three patients (Figures 3E, 4A, 3B). This finding is concordant with an article published in India. Today, noting that ignorant villagers were warned by branders that the patient would die if taken to a doctor [10].

Our study, involving 15 adult patients aged 21 to 58 years, documents the use of branding for both acute and chronic illnesses, with a predominance of male patients (12, 80%) and three female patients (20%), all from rural backgrounds. In most cases, branding was advised by untrained individuals or family members, and four patients reported paying for the procedure. Although no extensive studies on therapeutic branding in adults exist in the current medical literature, a few isolated case series on children suggest a disturbing continuity of the practice across age groups. A relevant study conducted in Andhra Pradesh [11] documented branding in seven children aged 4 to 14 years who presented with annular hyperpigmented scars on the dorsal forearms. These marks were discovered incidentally during outpatient visits for unrelated complaints. Upon detailed inquiry, the parents admitted that branding was performed as a traditional treatment for jaundice, using heated circular metal coils by local healers. While both studies underline the deeply rooted cultural reliance on branding, they differ in terms of clinical presentation, detection, and outcomes.

Branding is a criminal offence under the Indian Penal Code-324 [12]. The prevalence of these superstitious practices during a period of advancing medical technology calls for more vigorous efforts in health education and the provision of better health services for the rural population. Stringent laws should be enforced to ban this harmful practice [13]. There are several limitations of this study. First, this is a retrospective, single-center study and may not reflect the broader prevalence or true incidence. Recall bias and small sample size are other limitations. Photographic documentation and assessment of scars were observational without objective grading.

## Conclusions

This study provides important insights into the practice of therapeutic branding, highlighting its persistence in certain communities and its serious clinical implications. The analysis revealed that branding is often performed on individuals from rural, low-resource settings and is typically used for conditions such as jaundice and chronic pain. The practice was associated with significant morbidity, including diagnostic delays, secondary infections, and, in some cases, adverse outcomes in otherwise manageable conditions. Results of this study indicate an urgent need for targeted health education, community outreach, and strict enforcement of legal measures to eliminate this inhuman practice.
